# Vestibular disorders and monoamine neurotransmitters: Current evidence

**DOI:** 10.1016/j.isci.2026.116401

**Published:** 2026-06-23

**Authors:** Yujie Liu, Linglin Zhou, Xingxing Chen, E. Tian, Yisheng Lu, Peixia Wu, Sulin Zhang, Jun Wang

**Affiliations:** 1 Department of Otorhinolaryngology, Head and Neck Surgery, The First Affiliated Hospital, Jiangxi Medical College, Nanchang University, Nanchang, China; 2 Jiangxi Medical College, Nanchang University, Nanchang, China; 3 School of Nursing, Jiangxi University of Chinese Medicine, Nanchang, China; 4 Department of Otorhinolaryngology, Tongji Hospital, Tongji Medical College, Huazhong University of Science and Technology, Wuhan, China; 5 Department of Physiology, School of Basic Medicine, Huazhong University of Science and Technology, Wuhan, China; 6 ENT Institute and Otorhinolaryngology Department of Eye and ENT Hospital, State Key Laboratory of Medical Neurobiology and MOE Frontiers Center for Brain Science, Fudan University, Shanghai, China; 7 Department of Otorhinolaryngology, Union Hospital, Tongji Medical College, Huazhong University of Science and Technology, Wuhan, China

**Keywords:** health sciences, medicine, medical specialty, otorhinolaryngology, natural sciences, biological sciences, neuroscience

## Abstract

The vestibular system, a crucial sensorimotor apparatus for balance and spatial orientation, is regulated by neurotransmitters. Despite its essential function, the intricacies of neurotransmission within this system, particularly regarding monoamine neurotransmitters, remain not fully explained. A variety of vestibular disorders and therapeutic interventions are associated with balance regulation, underscoring the significance of understanding the neurotransmission in the vestibular system. This comprehensive review offers an overall insight into the role of monoamine neurotransmitters, including serotonin (5-HT), histamine, dopamine, and norepinephrine (NE), in the vestibular compensation following peripheral vestibular dysfunction. By examining the existing literature and integrating recent findings, monoamine neurotransmitters are closely associated with vestibular disorders, and their modulators have been used in the treatment of such conditions. Based on the vestibular dysfunction, we highlight the expression and functional significance of these neurotransmitters in the early vestibular compensation. Future research will further explore the role of neurotransmitters and their receptors in vestibular compensation, providing new ideas for the development of more effective treatment strategies.

## Graphical abstract

**Figure:**
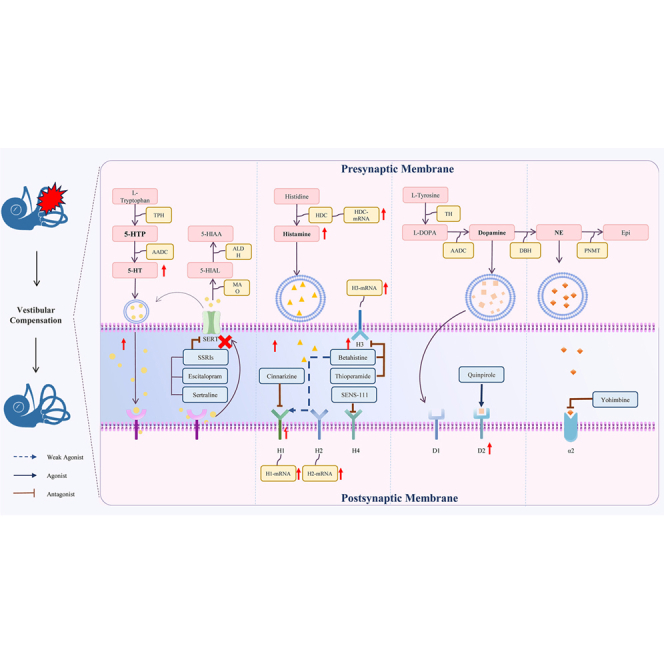

## Introduction

The vestibular system is a key sensory system for human balance and spatial orientation, which consists of peripheral vestibular organs, vestibular nuclei and vestibular pathways.^1^ The vestibular organs are located in the bony labyrinth of the inner ear, including the semicircular canals and otolith organs (utricle and saccule), of which the semicircular canals mainly sense the rotational movement of the head, while the otolith organs are sensitive to linear acceleration and gravity changes.^2^^,^^3^^,^^4^^,^^5^ Through exquisite biomechanical transduction, these structures convert head movements into neural signals, which are transmitted to the brain for processing and analysis to maintain body balance and stability.^6^ Disruptions in this intricate process, however, can lead to a variety of vestibular disorders, highlighting the importance of understanding the underlying mechanisms and developing effective compensatory strategies.^7^

Vestibular dysfunction, resulting from such disruptions, is primarily manifested as dizziness/vertigo, balance disorders, and blurred vision, etc. Common vestibular diseases, including benign paroxysmal positional vertigo (BPPV), Meniere’s disease (MD), often present with additional complications, such as anxiety, depression, and cognitive decline.^8^ Vestibular compensation refers to a neuroplastic process mediated by the central nervous system, through which the brain restores postural stability and spatial orientation following unilateral vestibular dysfunction (e.g., damage to the inner ear or vestibular nerve).^9^ Early vestibular compensation involves plastic events such as immediate-early genes, inflammatory factors, neurotrophic factors, neurotransmitters, ion channels, hormones, and neurogenesis.^10^^,^^11^ During the vestibular compensation process, neurotransmitters participate in the regulation of the body’s balance by affecting the excitability and inhibitory balance of vestibular neurons, especially monoamine neurotransmitters, such as dopamine, norepinephrine (NE), 5-hydroxytryptamine (5-HT; serotonin), and histamine.^12^^,^^13^ Notably, many modulators of these monoamine neurotransmitters—such as antipsychotics, antidepressants, anxiolytics, and anti-Parkinsonian drugs—are already widely used in the treatment of other neurological disorders.^14^^,^^15^^,^^16^ Nevertheless, the interplay between vestibular pathologies and monoamine neurotransmitters remains underexplored and warrants further investigation.

In this review, we aim to explore the link between vestibular diseases and monoamine neurotransmitters, their roles in vestibular compensation mechanisms, and the therapeutic potential of modulating these neurotransmitters. We provide a systematic overview of four major monoamine systems—serotonin, histamine, dopamine, and NE—synthesizing both clinical pharmacological evidence and preclinical mechanistic studies. Selective serotonin reuptake inhibitors have a significant effect on controlling vertigo in patients with MD, offering new insights for clinical treatment^17^; the modulation of histamine receptors offers new therapeutic avenues for the treatment of BPPV^18^; the balance of dopamine and NE affects the integration and regulation of vestibular signals, providing a scientific foundation for the development of novel treatment methods. It is expected that through a deeper understanding of the neurobiological basis of vestibular compensation, new directions and ideas will be provided for the design of more effective rehabilitation programs and drug treatment strategies, as well as future clinical practice and research.

## Methods

**Figure 1:**
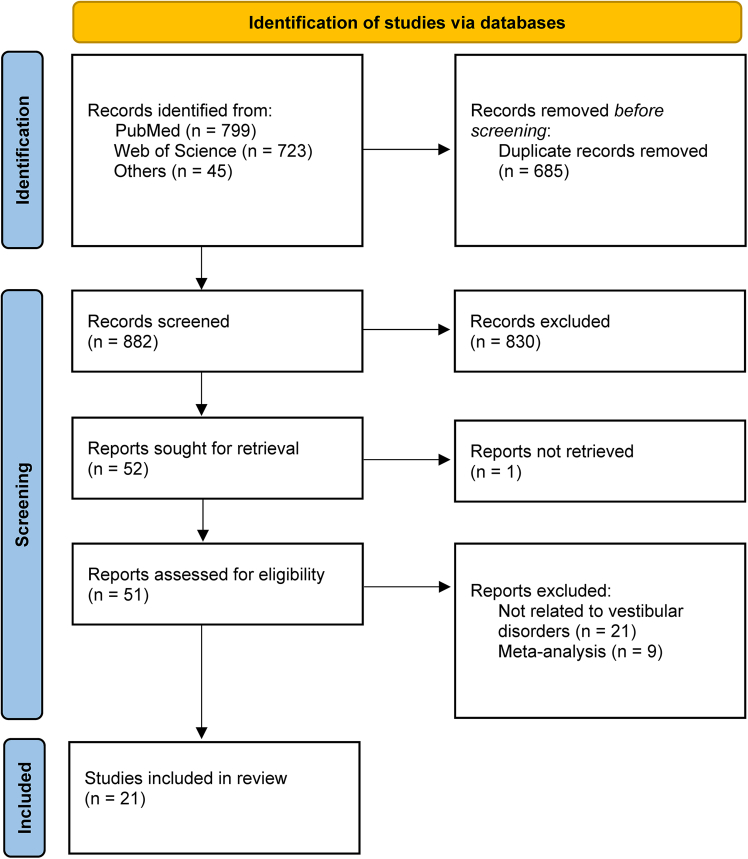
PRISMA flow diagram of the literature search and screening process

A systematic literature search was conducted using PubMed, Web of Science, and Google Scholar databases for articles published up to December 2025. The search keywords included combinations of the following terms: “vestibular disorders,” “vestibular compensation,” “unilateral labyrinthectomy,” “serotonin,” “5-HT,” “histamine,” “dopamine,” “norepinephrine,” “noradrenaline,” “monoamine neurotransmitters,” “vertigo,” “Meniere’s disease,” and “benign paroxysmal positional vertigo.” Only peer-reviewed articles written in English were included. Additional relevant references were identified by manually screening the reference lists of included articles. The literature screening process is summarized in a PRISMA flow diagram (Figure 1). This is a narrative review, not a formal systematic review or meta-analysis. The PRISMA flow diagram and structured search strategy were used solely to enhance transparency and reproducibility of the literature retrieval process.

## Monoamine neurotransmitters in vestibular disorders

### 5-HT (serotonin)

5-HT is an important monoamine neurotransmitter that is found in approximately 1%–2% of serotonergic neurons in the central nervous system. It plays a crucial role in various brain functions, including sleep-wake cycles, cognition, emotion, sexual function, thermoregulation, and food intake.^19^^,^^20^ 5-HT also regulates diverse neurobiological processes, including neurite outgrowth, dendritic spine morphology, neuronal circuit formation, synaptic transmission, and synaptic plasticity.

This regulatory activity is primarily mediated by distinct 5-HT receptors, which are structurally classified into 7 subfamilies comprising 14 subtypes.^21^ Within the vestibular system, several receptor subtypes have been identified in the vestibular nuclear complex.^22^^,^^23^ For instance, 5-HT1A, 5-HT1B, 5-HT2A, and 5-HT7 receptors are all expressed in the vestibular nuclei, and activation of the 5-HT1A receptor in the medial vestibular nucleus (MVN) attenuates synaptic transmission and impacts vestibular-related motor function.^24^^,^^25^ In contrast, the roles of other 5-HT receptor subtypes—such as 5-HT2C, 5-HT3, and 5-HT4—in vestibular compensation remain largely unexplored.^26^^,^^27^^,^^28^ While these receptors are well characterized in other central nervous system functions, including mood regulation, gastrointestinal motility, and nausea, their specific contributions to vestibular processing and post-lesion recovery warrant further investigation.^29^^,^^30^^,^^31^^,^^32^

**Figure 2:**
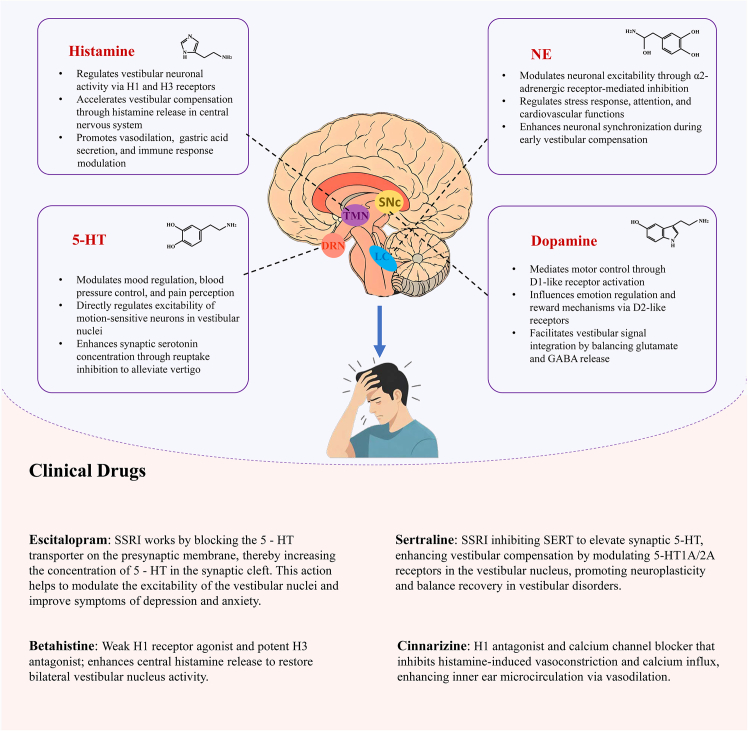
The functions and pharmacological applications of monoamine neurotransmitters in common vestibular diseases BPPV, benign paroxysmal positional vertigo; MD, Meniere’s disease; SSRI, selective serotonin reuptake inhibitor; GABA, gamma-aminobutyric acid.

Selective serotonin reuptake inhibitors (SSRIs) are well tolerated, easy to prescribe, and have low complication rates. They’ve also been shown to reduce dizziness in patients with various psychiatric conditions, including those with peripheral vestibular disorders and migraines^17^^,^^33^^,^^34^ (Figure 2; Table 1). A prospective pilot study by Simon et al. on 5 chronic vestibular dysfunction patients receiving 12-week fluoxetine (20–60 mg/d) demonstrated that while vestibular physiological parameters showed limited improvement, functional disability (dizziness handicap inventory [DHI] trend *p* = 0.07) and anxiety sensitivity (ASI significant reduction *p* < 0.05) were markedly alleviated.^34^ These findings suggest the 5-HT system may enhance central psychological compensation, potentially through modulating vestibulo-limbic network interactions, rather than directly repairing peripheral deficits. This provides preliminary evidence that SSRIs facilitate neural plasticity-mediated compensation by optimizing serotonin-regulated adaptive processing of vestibular signals. Patients with MD accompanied by anxiety and depression were administered escitalopram (10 mg/day), with a continuous treatment period of 12–24 months, showing a significant improvement or even disappearance of vertigo symptoms. On the other hand, MD patients without anxiety and depression, who took sertraline (50 mg/day), did not experience any vertigo symptoms during the one-year follow-up period after six months of continuous treatment.^17^

**Table 1:** An overview of the utilization of monoamine neurotransmitter modulators in the management of vestibular pathologies

Author, year	Study type	Patients	Monoamine modulators	Main findings
Goto et al., 2014^35^	case reports	MD (*n* = 3)	sertraline (50 mg/day), 6 months	in all three patients, vertigo attacks were completely controlled or significantly improved by the treated with sertraline.
Staab et al., 2002^33^	retrospective	(*n* = 60) including: (1) psychogenic dizziness (2) dizziness due to a neurotologic condition (3) idiopathic dizziness	SSRIs, the modal daily dose of sertraline is 50 mg (range 25–150 mg per day). fluoxetine 5–60 mg per day, paroxetine and citalopram 5–40 mg per day. All patients received SSRI treatment for at least 20 weeks.	SSRIs effectively relieved dizziness and improved psychiatric symptoms in a significant proportion of patients, with better outcomes observed in those with coexisting peripheral vestibular conditions or migraines. However, about 25% of patients could not tolerate SSRIs due to adverse effects, highlighting the need for controlled trials to confirm these findings.
Staab et al., 2004^36^	prospective	subjective dizziness (*n* = 20)	sertraline (median daily dose = 100 mg),16 weeks	sertraline significantly reduced dizziness in 55% of patients without active neurotologic illness, with a 73% positive response rate among completers.
Simon et al.^34^	prospective	5 patients (4 male, 1 female) with inner ear vestibular dysfunction and anxiety symptoms (*n* = 5)	fluoxetine (20–60 mg/day), 12 weeks	fluoxetine reduced anxiety and dizziness-related impairment significantly, with large effect sizes, but showed inconsistent vestibular/balance improvements.
Parfenov et al.^37^	prospective	vestibular vertigo (*n* = 309)	betahistine (48 mg/day, 2-month treatment + 2-month follow-up)	betahistine significantly reduced vertigo severity (74.1% good/excellent response) and attack frequency (median 8→0/month), with sustained effects post-treatment. Improvements likely mediated through vestibular compensation mechanisms. Supports betahistine as effective long-term therapy for vestibular vertigo in real-world settings.
Kiroglu et al.^17^	clinical report	MD (*n* = 12)	escitalopram (10 mg/day), 12∼24months, 2 years	escitalopram eliminated vertigo attacks in all 12 patients with Meniere’s disease and generalized anxiety disorder, indicating a 100% control rate of vertigo attacks.
Maslovara et al., 2012^18^	prospective	BPPV (*n* = 96) Control (*n* = 40)	betahistine chloride (24 mg/day, bid), 8 weeks	after one week, 86.96% of patients in the pharmacotherapy group and 93.33% in the rehabilitation group had negative Dix-Hallpike test results, indicating a more rapid recovery in the rehabilitation group. By the end of the eight-week treatment, 95.65% in the pharmacotherapy group and 97.78% in the rehabilitation group showed negative results, demonstrating a more complete recovery in the latter.
Scholtz et al., 2019^38^	prospective	peripheral vestibular vertigo of various origins (*n* = 306)	the fixed combination of cinnarizine and dimenhydrinate (3times/day, 4weeks)	the fixed combination of cinnarizine 20 mg and dimenhydrinate 40 mg showed superiority over betahistine 16 mg, reducing vertigo symptoms more effectively, with a significant mean vertigo score reduction of 0.395 compared to 0.488 for betahistine after 4 weeks of treatment.
Rizk et al.^39^	prospective	MD(*n* = 40)	venlafaxine (37.5 mg, daily for 8 weeks) Placebo (daily for 8 weeks)	there is no significant difference between venlafaxine and placebo in reducing vertigo episodes or improving quality-of-life metrics. Longitudinal follow-up suggested sustained improvement in some patients’ post-study.

MD, Meniere’s disease; SSRIs, Selective serotonin reuptake inhibitors; BPPV, benign paroxysmal positional vertigo; DHI, Dizziness Handicap Inventory.

Regarding the mechanisms of action of 5-HT reuptake inhibitors in treating vertigo disorders, the following hypotheses have been proposed^33^^,^^35^^,^^40^^,^^41^: (1) by inhibiting the reuptake of serotonin, the concentration of 5-HT in the synaptic cleft is increased, which may lead to a reduction in anxiety and depressive levels, thereby controlling the onset of vertigo. (2) Serotonin that is centrally generated and released may directly modulate the excitability of motion-sensitive neurons in the vestibular nuclei, inferior olivary nuclei, medial cerebellar nuclei, and brainstem autonomic centers, which could facilitate vestibular compensation.

### Histamine

Histamine is a biogenic amine that is extensively present in both the central nervous system and peripheral tissues, playing a pivotal role in a spectrum of physiological functions. These include the regulation of sleep cycles, cognitive processes, emotional balance, appetite control, and immune system responses.^42^ Within the central nervous system, histamine is predominantly produced and released by histaminergic neurons that originate from the tuberomammillary nucleus (TMN) located in the posterior hypothalamus.^43^ Histamine exerts its effects primarily through its receptors, which are categorized into four types: H1, H2, H3, and H4. The H1 receptor is mainly involved in vasodilation, increased vascular permeability, smooth muscle contraction, and acceleration of heart rate.^44^^,^^45^ In allergic reactions, the activation of the H1 receptor is a major cause of symptoms, such as itching, sneezing, and rhinorrhea.^46^^,^^47^ The H2 receptor promotes gastric acid secretion and vasodilation. It is associated with cell proliferation and wound healing.^48^ The H3 receptor primarily modulates the release of neurotransmitters in the central nervous system and is related to various central nervous system dysfunctions.^49^ Lastly, the H4 receptor is engaged in the modulation of immune responses, particularly within immune cells.^50^^,^^51^

Previous studies have indicated that in clinical treatment, histamine H1 receptor antagonists, such as betahistine and cinnarizine, can treat vertigo disease^52^^,^^53^ (Figure 2; Table 1). Betahistine, which functions as a weak agonist at the histamine H1 receptor and a potent antagonist at the H3 receptor, has been shown to effectively alleviate vertigo symptoms and facilitate vestibular compensation based on clinical evidence.^54^^,^^55^ Its effects in reducing spontaneous nystagmus (SN) and promoting static vestibular compensation have been confirmed in animal experiments.^56^^,^^57^ Clinically, the combination of betahistine with vestibular rehabilitation therapy significantly improves the quality of life of patients with vestibular disorders and reduces the risk of falls,^52^ which may be achieved by inducing histamine release in the central nervous system, activating MVN neurons, and thus accelerating the recovery of imbalanced firing in bilateral MVN. Some researchers believe that betahistine has dose-dependent effects, and long-term administration of high-dose oral betahistine can effectively improve intractable dizziness in patients with poor vestibular compensation.^54^^,^^56^^,^^58^ A systematic review by Murdin et al. included 17 randomized controlled trials, and the analysis showed that the proportion of patients who reported an overall reduction in vertigo symptoms with betahistine treatment was significantly higher than that of the placebo group.^54^ Cinnarizine is an antihistamine drug, and in Scholtz’s prospective study, vertigo patients treated with 20 mg cinnarizine and 40 mg dimenhydrinate saw a 61% drop in mean vertigo scores (MVS) over 8.4 weeks, with 22.1% becoming symptom free. Side effects like nausea and tinnitus also decreased. This treatment was more effective and safer than 16 mg betahistine for vestibular disorders over 4 weeks.^53^

In the study by Sinisa et al., 96 patients with a score of ≥40 on the DHI test for BPPV were included.^18^ Group I received treatment with betahistine hydrochloride medication, while group II underwent Epley maneuver rehabilitation therapy. At the initial follow-up one week post-treatment, 86.96% of the patients in group I and 93.33% in group II had a negative Dix-Hallpike test result. After 8 weeks of treatment, the second follow-up revealed that 95.65% of group I and 97.78% of group II had negative test results. Both groups demonstrated significant improvement after eight weeks; however, statistical analysis showed that patients who underwent physical therapy and rehabilitation (group II) experienced a more rapid and complete recovery compared to those who were treated with betahistine hydrochloride medication (group I). Further research is warranted to delve into the role of histamine receptor-related drugs in treating BPPV and to determine how best to integrate medication with rehabilitation therapy, with the aim of enhancing treatment efficacy and the quality of life for BPPV patients.^18^

Parfenov et al. conducted a multinational prospective observational study (VIRTUOSO) that substantiates betahistine’s capacity to potentiate vestibular compensation mechanisms.^37^ Their findings demonstrated that 48 mg/day betahistine administration over 60 days induced significant reductions in vertigo severity and attack frequency (median monthly episodes reduced from 8 to 2, *p* < 0.001). Crucially, the therapeutic effects exhibited progressive enhancement during the 2-month post-treatment follow-up, with 74.1% of patients maintaining good-to-excellent clinical responses and median attack frequency further declining to 0 (*p* < 0.001 vs. end of treatment). This sustained efficacy trajectory suggests betahistine may facilitate long-term neuroplastic adaptations in central vestibular pathways.

A recent nationwide retrospective cohort study was conducted using electronic health records of 78,452 vestibular disorder patients from 2010 to 2020, revealed that meclizine, a histamine H1 receptor antagonist, exhibits significantly less interference with vestibular compensation mechanisms compared to GABAergic agents.^59^ As a selective H1 histamine receptor antagonist, meclizine was associated with 41% reduced fall risk (adjusted hazard ratio [HR] 0.59, 95% confidence interval [CI] 0.52–0.67) during 90-day follow-up in propensity score-matched analyses. The observational study implemented inverse probability treatment weighting to control confounding variables including age, comorbidities, and baseline vestibular function. Pharmacodynamic profiling suggests that meclizine’s preferential blockade of histaminergic pathways (Ki = 8.3 nM for H1 receptors) may preserve GABAergic plasticity in MVN, which could explain its milder compensation disruption. This finding warrants mechanistic investigation into histamine-mediated modulation of synaptic reorganization during vestibular compensation, particularly through H1 receptor-dependent neuroplasticity pathways (Table 1).

### Dopamine and NE

Dopamine is primarily produced by dopaminergic neurons in the substantia nigra pars compacta and the ventral tegmental area of the brain, and it is transmitted to other regions, such as the prefrontal cortex, striatum, and hippocampus via neuronal axons.^60^ Dopamine exerts its effects by binding to its receptors, which include the D1-like receptors (D1 and D5) and the D2-like receptors (D2, D3, and D4). D1-like receptors are mainly associated with enhancing neurotransmission and promoting motor control, while D2-like receptors are involved in regulating emotions, appetite, and reward mechanisms. The balance of dopamine is crucial for maintaining normal physiological and psychological states, and its abnormal levels are closely related to neuropsychiatric disorders, such as Parkinson’s disease, schizophrenia, and addiction.^61^^,^^62^ The D1-like receptors typically facilitate the release of glutamate, an excitatory neurotransmitter, while the D2-like receptors are associated with the inhibition of GABA release, a major inhibitory neurotransmitter. Abnormal levels of dopamine are associated with these disorders, likely due to their disruption of neurotransmitter balance in the brain, which may affect key brain functions, such as mood, movement, and reward^63^ (Figure 2).

NE is primarily produced by the locus coeruleus in the brainstem, and its receptors are divided into two major categories: alpha-adrenergic receptors and beta-adrenergic receptors. The alpha-adrenergic receptors are further subdivided into two subtypes, α1 and α2. Activation of α1 receptors is typically associated with vasoconstriction, increased blood pressure, and excitation of smooth muscle, while α2 receptors play a role in regulating the auto-inhibition of NE release and decreasing heart rate.^64^ The beta-adrenergic receptors are divided into three subtypes, β1, β2, and β3. β1 receptors mainly affect the heart, increasing heart rate and myocardial contractility^65^; β2 receptors promote vasodilation and metabolic activity^66^; β3 receptors have a direct effect on the activation of adult hippocampal progenitor cells.^67^ By activating various types of receptors, NE plays a crucial role in regulating stress responses, attention, mood, blood pressure, and heart rate (Figure 2).

## Vestibular compensation and monoamine neurotransmitters

### Changes in 5-HT levels in the unilateral vestibular dysfunction model

**Figure 3:**
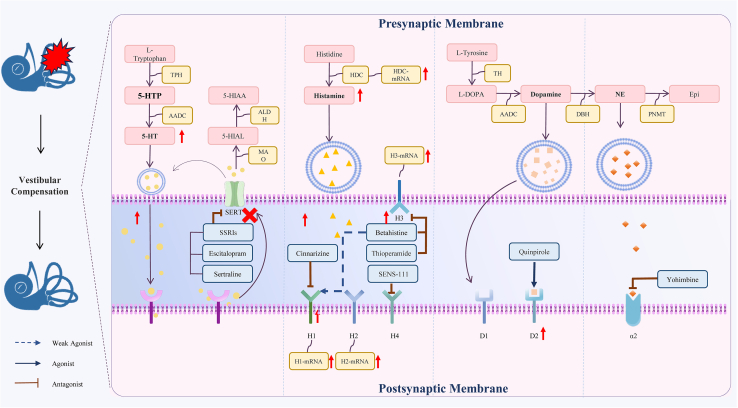
Molecular pathways of monoaminergic regulation in vestibular compensation 5-HT is derived from L-tryptophan by tryptophan hydroxylase (TPH) and aromatic L-amino acid decarboxylase (AADC), stored presynaptically, and reabsorbed via the serotonin transporter (SERT). Catabolism by monoamine oxidase (MAO) yields 5-hydroxyindoleacetic acid (5-HIAA). Histamine is synthesized from histidine via histidine decarboxylase (HDC) and acts postsynaptically through H1, H2, and H4 receptors. Its degradation is mediated by histamine N-methyltransferase (HNMT). Dopamine is converted to norepinephrine (NE) by dopamine β-hydroxylase (DBH); NE can be further converted to epinephrine by phenylethanolamine N-methyltransferase (PNMT). α2-Adrenergic receptors regulate NE release, synergizing with histamine in anti-inflammatory pathways.

Considering the rich serotonergic input that the MVN receives from the dorsal raphe nuclei (DRNs), serotonin (5-HT) is exceptionally well suited to function as a neuromodulator in the vestibular system, playing a crucial role in the regulation and execution of postural and bodily movements.^68^ Research has demonstrated that 5-HT receptors are critical for central nervous system plasticity. The 5-HT1A, 5-HT1B, 5-HT2A, and 5-HT7 receptors are all expressed in the vestibular nuclear complex; however, their roles in vestibular compensation remain unclear, and limited research has addressed this mechanism. Cransac et al., utilized high-performance liquid chromatography (HPLC) to evaluate 5-HT levels in rat cochlear nuclei. They found lower 5-HT concentrations in these nuclei compared to the dorsal raphe nucleus (RD), with higher levels in the anterior ventral cochlear nucleus (AVCN). The metabolic ratio of 5-HIAA to 5-HT indicated higher serotonergic activity in dorsal nuclei, suggesting a differential role for 5-HT in auditory processing across cochlear subnuclei.^69^ Wang et al. performed unilateral labyrinthectomy (UL) on rats and used HPLC to analyze the expression levels of monoamine neurotransmitters such as 5-HT in the MVN at different time points (4 h, 8 h, 1 day, 2 days, 4 days, and 7 days) after UL. As a result, the levels of 5-HT and NE in the ipsilateral MVN significantly increased within 4 days after UL, reaching peak values at 1 day and 2 days, respectively,^70^ suggesting that UL induced the activation of the 5-HT and NE systems. Another study by Zhang et al. performed unilateral horizontal semicircular canal occlusion (USSCO) on guinea pigs and monitored 5-HT levels in the MVN at 1, 3, and 5 days postoperatively using HPLC-ECD. They found a significant rise in 5-HT levels to 448.85 ± 24.56 nM in the USSCO group 24 h post-surgery, compared to the control group’s 356.37 ± 15.69 nM. This increase gradually returned to baseline levels within 3–5 days post-surgery, suggesting that USSCO may have activated the 5-HT system.^40^ In addition, Zhai et al., constructed an animal model of vestibular function damage by injecting gentamicin into the rat’s middle ear cavity and found that 3 days after administration, the concentrations of NE and 5-HIAA in the operated side MVN, LC, and DRN were significantly higher than those in the control group^41^ (Figure 3; Table 2).

**Table 2:** Summary of monoamine neurotransmitters in vestibular dysfunction animal models

Author, year	Model	Operation	Measures	Main findings
Cransac et al.^69^	Sprague-Dawley rats	three groups were injected intraperitoneally with a-methyl-paratyrosine methyl ester (AMPT, 250 mg/kg i.p.) 1, 2 or 3 h before killed. cervical dislocation, serial coronal sections	HPLC-ECD	5-HT and 5-HIAA levels were by far lower in the CN than in RD. The AVCN had a greater 5-HT level than DCN + PVCN; however, 5-HT utilization assessed from the ratio 5-HIAA/5-HT was slightly higher in the dorsal nuclei.
Cransac et al.,1995^69^	Sprague-Dawley rats	three groups were injected intraperitoneally with a-methyl-paratyrosine methyl ester (AMPT, 250 mg/kg i.p.) 1, 2 or 3 h before killed. cervical dislocation, serial coronal sections	HPLC-ECD	in the MVN, the concentrations of 5-HT were almost as high as in the LC but by far smaller than in the RD. 5-HIAA levels and the ratio 5-HIAA/5-HT were lower in MVN than in LC and RD.
Cransac et al.,1996^71^	DA-HAN rats, aged 4, 21 and 24 months	cervical dislocation, serial coronal sections	HPLC-ECD	in older rats, the main noradrenergic changes were a decrease of NE content with an increase of the MHPG/NE ratio in MVN and a selective NE increase in AVCN. 5-HT and 5-HIAA levels were increased in all the brainstem nuclei except raphe dorsalis. DA and DOPAC remained unchanged.
Drago et al.^72^	Sprague–Dawley rats	UL	open field test, Rotorod Test	high doses of the D2 receptor agonist quinpirol (2 or 4 mg/kg/day) facilitate vestibular compensation in aged rats after UL, whereas the D2 receptor antagonist sulpiride (4 mg/kg/day) inhibits it.
Kuruma et al., 2003^73^	Wistar rats	cisplatin injecting in the right ear to induce inner ear damage. After 1–2 weeks, rats were perfused for brain specimen preparation.	immunohistochemical staining	cisplatin rats showed significant reduction in TRH neurons in the ipsilateral raphe nucleus, DA neurons in the ipsilateral hypothalamus, and NE neurons in the ipsilateral locus coeruleus and ventral pons. Saline rats showed no such differences.
Lozada et al.^74^	Rats	UL	probe H3X	the levels of H3 receptor mRNA significantly increased 24 h post-lesion and subsequently decreased at 48 h and one week post-lesion.
Goddard et al., 2008^75^	Rats	BVD	western blot	BVD rats showed a significant decrease in TH expression in the frontal lobes, a significant decrease in SERT expression in the frontal lobes and CA1 region, and a significant increase in TryH expression in the frontal lobes, CA2/3, and dentate gyrus, while TryH expression decreased in the entorhinal cortex. No significant changes were observed in DH or DAT expression.
Zhou et al.^76^	Sprague-Dawley rats,	UL	RT-qPCR, western blot immunofluorescence	on the ipsi-lesional flocculus the H1, H2 and H3 receptors mRNA and the protein increased significantly on the 1st and 3rd day, with compare of sham controls and as well the contralateral side of UL. However, on the 7th day after UL, this expression returned to basal level.
Zhang et al.^40^	Guinea pigs	USSCO, lateral horizontal semicircular canal occlusion	HPLC-ECD	unilateral horizontal semicircular canal occlusion could increase the 5-HT level in MVN.
Zhai et al.^41^	Sprague-Dawley rats	gentamicin injection	HPLC	within MVN, the concentration of NE and 5-HIAA of GT-3d group increased significantly compared with the control group, and the concentration of DA and DOPAC increased markedly compared with the sham surgery group.
Chen et al.^77^	Sprague-Dawley rats	UL	western blot, Retrograde tracing, Immunofluorescence	the histamine H1 receptor played a significant role in facilitating vestibular compensation, and that its blockade in the MVN significantly delayed the recovery from both static and dynamic vestibular symptoms following UL. Additionally, the H1 receptor appeared to be involved in the therapeutic effects of betahistine.
Wang et al.^70^	Sprague-Dawley rats	UL	HPLC	compared to the sham control group, the levels of 5-HT and NE in the ipsilesional MVN of the UL group were significantly elevated within 4 days after UL, peaking on 1 day and 2 days, respectively. The levels of DA showed an increasing trend at different time points up to 7 days post-UL, while histamine levels significantly increased only at 1day post-UL.

AMPT, α-methyl-paratyrosine; HPLC-ECD, high-performance liquid chromatography and electrochemical detection; CN, cochlear nuclei; HPLC-CL, high-performance liquid chromatography coupled with gold nanoparticle-initiated chemiluminescence; RD, dorsal raphe nucleus; AVCN, anteroventral cochlear nucleus; DCN, dorsal cochlear nucleus; PVCN, posteroventral cochlear nucleus; LC, locus coeruleus; MVN, medial vestibular nucleus; NE, norepinephrine; MHPG, 3-methoxy,4-hydroxyphenylglycol; BVD, bilateral surgical vestibular deafferentation; DA, dopamine; DOPAC, 3,4-dihydroxyphenylacetic acid; UL, unilateral labyrinthectomy; USSCO, unilateral single semicircular canal occlusion.

Collectively, these findings suggest that the 5-HT system may enhance central psychological compensation through modulating vestibulo-limbic network interactions, rather than directly repairing peripheral deficits.

It is worth noting that not all serotonergic agents have shown consistent efficacy. In a recent randomized controlled trial, Rizk et al. found that venlafaxine—a serotonin-NE reuptake inhibitor—did not significantly reduce vertigo episodes or improve quality of life in patients with MD compared with placebo.^39^ This discrepancy may be attributable to several factors, including the lower dose used (37.5 mg/day), the relatively short treatment duration (8 weeks), the strong placebo effect known in MD, and the fact that the study enrolled patients regardless of comorbid anxiety or depression.^39^ These findings underscore the importance of patient selection, drug choice, and dosing strategies in future studies, and do not necessarily negate the therapeutic potential of serotonergic modulation in appropriately selected patient subgroups.

### Histamine receptors involved in vestibular compensation

The central histaminergic system, which is exclusively derived from the TMN of the hypothalamus, has been long associated with the regulation of vestibular functions and the subsequent recovery process following vestibular damage.^77^^,^^78^^,^^79^ Brahim et al., found that in cats, 1 week after unilateral vestibular neurectomy (UVN), there was a significant increase in the expression of histidine decarboxylase (HDC) mRNA in the ipsilateral TMN, indicating an acute response to the vestibular injury.^80^ This increase was mainly observed in the TMN on the side of the injury, with less expression change on the non-injured side, suggesting that the asymmetry of vestibular input affects the histaminergic system. Three weeks later, the HDC mRNA expression levels in these cats decreased but remained higher than in the control group, indicating that the histaminergic system is still adjusting during the process of vestibular compensation. At three months, HDC mRNA expression returned to control levels in both TM nuclei, consistent with the complete recovery of behavioral function. Additionally, cats treated with the histamine H3 receptor antagonist thioperamide after UVN showed faster behavioral recovery, including improvements in SN, posture, and motor balance, indicating that histamine receptors play an important role in vestibular compensation.^80^^,^^81^

Zhou et al. found that in rats one day after UL, the mRNA and protein levels of H1, H2, and H3 receptors in the ipsilateral MVN increased.^76^ Chen et al. also performed UL on rats and found that the expression level of H1 receptors in the ipsilateral MVN significantly increased from 2 h to 4 days, returning to baseline levels after 7 days, and H1 receptors significantly weakened the promoting effect of betahistine, suggesting that histamine receptors are actively involved in vestibular compensation^77^ (Figure 3; Table 2).

According to Housley et al., using the semicircular canal of a frog, the biphasic triangular motion of the endolymph induced by a piezoelectric actuator was used to elicit afferent nerve responses from the semicircular canal.^82^ It was found that histamine at a concentration of 10^ˆ−7^ mol could promote the firing rate of vestibular nerve afferents, increasing by 13.4% ± 3.53%, and this promoting effect reached its maximum at a concentration of 10^ˆ−6^ mol. Additionally, the use of the histamine synthesis inhibitor α-fluoromethylhistidine (α-FMH) could dose-dependently reduce the firing of vestibular nerves, indicating that histamine may have an endogenous neuromodulatory role in the semicircular canal.^82^ In recent studies, vestibular injury was induced in rats by unilateral transtympanic injection of kainate, and the administration of 10 mg/kg of SENS-111 (a novel histamine H4 receptor antagonist) significantly reduced the frequency of SN in rats within 1 h after administration, indicating that blocking histamine H4 receptors can reduce the hyperactivity of vestibular neurons, thereby alleviating symptoms of vertigo.^83^

### Exploration of the role of dopamine in vestibular compensation

Imbalances in dopamine release in the central nervous system can lead to various manifestations, such as motor disorders, emotional abnormalities, attention and cognitive dysfunction. In the Lewis (LEW) rat mutant model (ci2/ci2), observed features such as, hair cell loss, deafness, vestibular dysfunction, lateral rotation, hyperactivity, and alterations in the nigrostriatal dopamine system suggest a link between the dopaminergic system and vestibular deficits, highlighting the system’s potential role in vestibular compensation.^84^ Autoradiographic results of dopamine receptors D1 and D2 in the midbrain striatum after unilateral/bilateral vestibular damage in rats show that the density of D1 and D2 receptors increased to varying degrees after vestibular damage, providing evidence that the dopamine system may be involved in vestibular compensation.^85^ Animal studies have investigated the potential of dopaminergic medications to enhance vestibular compensation. Treatment of rats after UL with dopamine receptor agonists (SKF38393 and quinpirole) or antagonists (SCH23390 and sulpiride) found that quinpirole (D2 receptor agonist) can promote the decrease in SN frequency, while sulpiride (D2 receptor antagonist) inhibits the decrease in SN.^86^ Drago et al. showed that in aged rats after UL, the use of quinpirole as a D2 receptor agonist at a dose of 4 mg/kg/day accelerated the decay rate of SN, improved motor ability and coordination, and significantly promoted vestibular compensation in aged rats. In contrast, the D2 receptor antagonist sulpiride inhibited this compensation process at the same dose.^72^ Deshetty et al. also found that in a mouse model, after experiencing rotation-induced motion sickness, the use of menthol (MNT) at a dose of 50 mg/kg reduced the expression levels of dopamine and dopamine D2 receptors (DRD2) in the striatum and brainstem, effectively alleviating the mouse’s motion sickness symptoms, improving its motor coordination ability, and significantly enhancing the mouse’s adaptability to motion sickness^87^ (Figure 3). The aforementioned results indicate that dopamine neurotransmission plays a role in the process of vestibular compensation after UL, but the underlying mechanism still needs to be elucidated.

### NE dynamics during vestibular compensation processes

In the vestibular system, NE modulates neuronal excitability and may play a crucial role in vestibular compensation. Wang et al. demonstrated that following unilateral vestibular injury, NE levels significantly increase in the ipsilateral vestibular nuclei, indicating that the NE system is activated in response to injury.^70^ This activation may enhance neuronal “sentience” and promote synchronized activity among neurons, thereby accelerating the recovery of vestibular function.

In a study by Licata et al., the effects of microiontophoretic NE on the firing rate of neurons in the vestibular complex were examined in anesthetized rats.^88^ The results showed that 85% of the neurons tested in the vestibular nuclei modified their background firing rate upon NE application, with 86% exhibiting a decrease in firing rate. The inhibitory effects were dose dependent, with maximal effects observed at an ejection current of 60 nA. These inhibitory responses were primarily mediated by α_2_-adrenergic receptors, as they were blocked by the α_2_-adrenergic antagonist yohimbine and mimicked by the α_2_-adrenergic agonist clonidine.^88^ These findings suggest that NE can directly influence the firing rate of vestibular neurons, predominantly through α_2_-adrenergic receptors. This modulation of neuronal activity by NE is consistent with its role in vestibular compensation, where it may help to reduce the background firing rate of vestibular neurons, potentially facilitating the recovery of balance function. The ability of NE to modulate neuronal excitability and responsiveness supports the hypothesis that it plays a crucial role in the early stages of vestibular compensation following peripheral vestibular lesions.

From a neurophysiological perspective, the role of NE is closely related to the “subjective sentience” of neurons. Di Mauro et al. pointed out that NE modulates neuronal excitability by regulating membrane permeability, allowing neurons to more sensitively detect the electrochemical state of their external environment.^89^ This “sentience” is particularly important during vestibular compensation, as neurons need to rapidly sense and respond to changes in their environment following injury to facilitate functional recovery. The increase in NE levels may enhance neuronal sentience, which could promote synchronized activity among neurons and contribute to the recovery of vestibular function.^89^ Moreover, the modulatory effects of NE may also be relevant to the recovery of cognitive functions. Following vestibular injury, patients not only experience impairments in balance function but may also suffer from cognitive deficits, such as inattention and emotional changes. The increase in NE levels may help restore these cognitive functions by modulating neuronal excitability, thereby facilitating the overall process of vestibular compensation (Figure 3).

### Interactions among monoamine neurotransmitter systems in vestibular compensation

Although 5-HT, histamine, dopamine, and NE are traditionally discussed as separate systems, accumulating evidence suggests that they do not operate in isolation during vestibular compensation. Schlicker et al. systematically reviewed that H3 heteroreceptors are distributed on serotonergic, dopaminergic, and noradrenergic neurons in the brain of various mammalian species, and the activation of these H3 heteroreceptors inhibits the release of the corresponding transmitters.^90^ Haas et al. (2008) further noted that H3 receptors act as inhibitory auto- and heteroreceptors, and that mutual interactions with other transmitter systems form a network that links basic homeostatic and higher brain functions.^91^ Thus, the histamine H3 receptor, by virtue of its dual function as both an autoreceptor and a heteroreceptor, serves as a central molecular hub for cross-regulation among monoamine systems.

Under normal physiological conditions, these monoamines exert clear modulatory effects on vestibular neuronal activity; however, whether multiple systems coordinately participate under pathological conditions remains unclear. Lozada et al.^74^ found significant plastic changes in histamine H3 receptor expression and binding in the rat vestibular nuclei following labyrinthectomy, and that betahistine, as an H3 receptor antagonist, can accelerate vestibular compensation, suggesting that H3 receptors may serve as a key node for crosstalk among monoamine systems.^74^ Bergquist et al. further confirmed that histamine directly inhibits GABA release via H3 receptors in the vestibular nuclei and also acts through indirect pathways.^92^ The observation that the H3 receptor antagonist thioperamide accelerates behavioral recovery during vestibular compensation in cats^80^^,^^81^ also supports this view. Zhai et al. reported that vestibular injury simultaneously elevated NE and 5-HIAA levels in the MVN, locus coeruleus and dorsal raphe nucleus.^41^ Similarly, Wang et al. simultaneously measured all four monoamines in the rat MVN after unilateral labyrinthectomy and found that all were significantly elevated but with distinct temporal dynamics, indicating that peripheral vestibular injury triggers a coordinated multi-system monoaminergic response.^70^ These findings suggest that each transmitter system contributes to the regulation of vestibular compensation with its own temporal dynamics.

Despite the evidence for monoamine interactions, systematic studies specifically investigating the interactions among these systems in the context of vestibular compensation remain very limited. Future research should pay attention to the dynamic interactions among multiple monoaminergic systems, which may provide new theoretical foundations for optimizing therapeutic strategies for vestibular disorders.

## Future direction

Although we have gained a preliminary understanding of the role of neurotransmitters in the process of vestibular compensation, the precise mechanisms still require further in-depth investigation (Table 3 and Table 4). In the present review, we have discussed serotonin and histamine in greater detail, reflecting the more extensive literature available for these two systems. By contrast, dopamine and NE are supported by relatively limited vestibular-specific evidence. This paucity of studies, rather than indicating lesser importance, highlights a particularly promising direction for future research. Accordingly, future studies should focus on elucidating the specific receptors and downstream signaling pathways that each monoamine neurotransmitter targets in the context of vestibular compensation, with special emphasis on the underinvestigated dopaminergic and noradrenergic systems. Additionally, it is essential to employ neuropharmacological, chemogenetic, and electrophysiological approaches to gain a deeper understanding of the neural circuitry interconnections between monoamine-associated brain regions and the vestibular nuclei. Furthermore, further investigate how these neuromodulators might be applied clinically, including the initiation of clinical trials to assess the levels of monoamine neurotransmitters and to investigate the impact of different monoamine modulators on vestibular disorders.

**Table 3:** Temporal profiles of monoamine neurotransmitter changes during early vestibular compensation after unilateral vestibular dysfunction

Neurotransmitter	Onset of increase	Peak time	Duration of elevation	Return to baseline	Trend description
5-HT	within hours post-UL	Day 1	∼4 days	Day 3–5	rapid sharp rise, then quick decline
Histamine	Day 1	Day 1	<24 h	Day 2	very narrow single peak
Dopamine	Day 1–2	not reached within 7 days	At least 7 days	>7 days	slow, sustained increase without clear peak
NE	within hours post-UL	Day 2	∼4 days	Day 7	rapid rise, peak at day 2, then gradual decline

Data were compiled from studies using high-performance liquid chromatography in rats and guinea pigs (Wang et al.^70^; Zhang et al.^40^; Chen et al.^77^; Zhou et al.^76^; Tighilet et al.^80^).

**Table 4:** Current knowledge and knowledge gaps regarding monoamine neurotransmitters in vestibular dysfunction

Neurotransmitter	What we know	What is missing
5-HT	5-HT_1_A, 5-HT_1_B, 5-HT_2_A, 5-HT_7_ receptors expressed in vestibular nuclei^24^^,^^25^ SSRIs reduce vertigo and anxiety in Meniere’s disease and chronic dizziness^33^^,^^35^^,^^40^^,^^41^ UL increases 5-HT level in ipsilateral MVN^70^ negative RCT with venlafaxine suggests importance of patient selection/dosing^39^	roles of 5-HT_2_C, 5-HT_3_, 5-HT_4_ receptors in vestibular compensation unexplored^93^ direct vs. indirect (limbic-mediated) mechanisms not dissected optimal dose, duration, and patient subgroups for serotonergic drugs unclear interaction with other monoamines during vestibular compensation remains speculative
Histamine	H_1_, H_2_, H_3_ receptor mRNA/protein increase in ipsilateral MVN after UL^77^ H_3_ heteroreceptors regulate release of 5-HT, DA, and NE^90^^,^^91^ H_3_ antagonist thioperamide accelerates behavioral recovery in cats^80^^,^^81^ betahistine improves vertigo and vestibular compensation^37^^,^^52^^,^^54^^,^^55^	translational human mechanistic studies lacking whether H_3_ effects on vestibular compensation are direct or indirect via other monoamines needs clarification interaction with rehabilitation therapy not fully optimized^18^
Dopamine	D_1_ and D_2_ receptors in striatum change after vestibular lesion^85^ D_2_ agonist quinpirole promotes, D_2_ antagonist sulpiride inhibits compensation after UL in rats^72^^,^^86^ dopamine levels in MVN show an increasing trend up to 7 days post-UL^70^	limited vestibular-specific evidence (much less than 5-HT/histamine) interaction with other monoamine systems not investigated
NE	NE levels increase in ipsilateral MVN, locus coeruleus, and raphe after UL^41^ NE modulates MVN neuronal firing mainly via α_2_-adrenergic receptors^88^	specific roles of α_1_, α_2_, β receptor subtypes in vestibular nuclei unknown no interventional studies targeting NE alone for vestibular disorders synergistic or additive effects with 5-HT or histamine unexplored clinical evidence for NE-targeting drugs inconclusive^39^

5-HT, 5-hydroxytryptamine; MVN, medial vestibular nucleus; UL, unilateral labyrinthectomy; DA, dopamine; NE, norepinephrine.

## Conclusion

This review summarizes the complex relationship between common vestibular diseases and monoamine neurotransmitters and explores the mechanisms of action of these neurotransmitters in the process of vestibular compensation (Figures 2 and 3). Monoamine neurotransmitters such as 5-HT, histamine, dopamine, and NE appear to influence vestibular compensation, possibly by regulating balance between excitation and inhibition in vestibular neurons. Specifically, 5-HT plays a role in mood regulation, blood pressure control, and pain perception through its diverse receptor subtypes and has shown potential in the treatment of vestibular diseases. For example, SSRIs have demonstrated significant effects in improving the vertigo symptoms and accompanying emotional disorders in patients with MD. In addition, histamine participates in the regulation of vestibular neuronal activity through its H1 and H3 receptors, and its agonists and antagonists have shown certain therapeutic effects in the treatment of vestibular disorders. Dopamine and NE are involved in the integration and regulation of vestibular signals by affecting the excitability of vestibular neurons, and their importance in vestibular compensation has been confirmed in animal models. These findings not only deepen our understanding of the neurobiological mechanisms underlying vestibular disorders but also offer critical insights for clinical intervention.

## Acknowledgments

This work was supported by grants from the 10.13039/501100020771Young Scientists Fund of the National Natural Science Foundation of China (82501420), Jiangxi Provincial Natural Science Foundation Youth Project (20252BAC200112), Jiangxi Health Commission's Special Research Fund for Talent Introduction & Cultivation (2026Y0027), and the First Affiliated Hospital of Nanchang University Clinical Research and Cultivation Project (grant no. YFYLCYJPY202437).

## Author contributions

J.W., P.W., and S.Z. conceived the project and developed its outline. Y. Liu and J.W. drafted the manuscript; Y. Liu, L.Z., X.C., E.T., Y. Lu, P.W., S.Z., and J.W. participated in data acquisition and table preparation; Y. Liu, L.Z., X.C., E.T., Y. Lu, P.W., S.Z., and J.W. reviewed and revised the manuscript. All co-authors contributed to the article and approved the final manuscript.

## Declaration of interests

The authors declare no competing interests.
